# Topics of Public Interest

**Published:** 1911-12

**Authors:** 


### TOPICS OF PUBLIC INTEREST

### A Buffalo Hospital for Alcoholics

By GEORGE H. McMICHAEL, M.D.
Buffalo, N. Y.

The legislature has passed, and the governor has signed a bill permitting any city of the first or second class to establish and maintain a hospital and industrial colony for alcoholics. The governing body of the institution is to consist of a board of seven members, five of whom are to be appointed by the mayor, and two of whom must be physicians.

The overseer of the poor of the city and a judge of the court which has jurisdiction over arrests for public intoxication are ex-officio members of the board. The five appointed members of the board, are to serve for five years.

The board of inebriety is required to maintain an office, which shall always be open, and at this office to keep a record of all persons arrested for public intoxication. Whenever any man, woman or child is arrested for public intoxication the officer in control of the police station to which the arrested individual has been taken is required at once to report the name and address of the accused to the office of the board of inebriety, which board must endeavor to ascertain if he has been arrested for public intoxication within the previous twelve months. If he has not, he may sign a request for immediate release, which release will be granted. A report of the case must be sent to the court having jurisdiction over such matters. In case the accused has been arrested on the charge of being drunk within twelve months, he may upon his own application, or upon the certificate of two examiners in lunacy, or upon the petition of a relative, or the overseer of the poor, be committed to the care of the board of inebriety for a period of from one to three years. The committing power is, of course any court of record.

The statute defines an inebriate as "a person who is incapable of properly conducting himself or his affairs, or is dangerous to himself or others, by reason of habits periodical, frequent or constant drunkenness, induced either by the use of alcoholic or other liquors, or of opium, morphine or other narcotic or intoxicating or stupefying substance."

The board of inebriety has power to parole, upon such terms as it may consider suitable, any patient committed to its care. The patient so paroled will be under the supervision of the board's probation officer until such time as the board considers that he may safely be released, or until the expiration of the maximum term for which he was committed. Any paroled patient who violates the terms of his parole may be arrested by order of the board and returned to its custody. If however, the board considers that the patient is an unsuitable person for further treatment, it may apply to the court which committed him to be released from further care of him. In that case the court may fine the offender or send him to the penitentiary. The amount of the fines and terms of imprisonment depend upon the number of times he has broken his parole, and the number of times he has been arrested for public intoxication.

Some fines may be paid in instalments, and they are to be paid to the board of inebriety. The new law makes provision for the treatment and care of non-residents if the board is willing to receive them. It provides for the appointment as superintendent of a physician who has had at least five years experience in the practice of medicine, and it defines his duties. In every case he is required to ascertain the financial position of the persons committed to the care of the board of inebriety and of the relatives who are liable for their support. When either patient or relatives are able to contribute toward the support of the former, the superintendent is given power to fix the sum per week that is to be paid. It must not, of course, exceed the cost of the patient's maintenance. The amount decided upon can be legally collected by the processes by which superintendents of the poor are allowed to recover such sums in similar circumstances.

The city in which the hospital and industrial colony are located is required to pay for patients who have no means, but non-residents' maintenance becomes a charge upon the civil division of the state upon which they would be a charge as poor persons. An important provision of the new law is that anybody who conveys alcoholic liquor or narcotic drugs to any place under the control of a board of inebriety, or who furnishes these liquors or drugs to any persons under the control of such a board without the written order of the superintendent or of a physician connected with the institution is guilty of a misdemeanor, and may be fined any sum between fifty and five hundred dollars, or may be imprisoned for not less than thirty days.

The committee appointed by Mr. Ansley Wilcox, President of the Charity Organization: Herbert B. Lee, Chairman; George B. Burd, Dr. Arthur W. Hurd, Dr. George H. McMichael, Newman Walbridge, all coöperating with Judge William R. Brennan and Francis Almy, Secretary of the C. O. S.

---

As the result of the sale of liquor containing wood alcohol, in Whitetown, Ind., four men are dead, one totally and two partially blind.

The Indiana State Medical Association held its annual meeting at Indianapolis, September 28 and 29. Advance reports render it probable that defense of members against malpractice suits, has been adopted, somewhat along the lines followed in New York and other states.

---

How Epidemics Originate. In November, a sailor suffering with malignant diphtheria was examined by a local physician and taken by the Charities Commissioner of Tonawanda to a Buffalo Hospital for treatment. P.S. The patient was taken in a trolley car on its regular trip. P.P.S. The matter is being investigated. We would like to comment on this item but we cannot think of anything to say that will not readily occur to our readers and, beside, there are certain regulations concerning the matter contained in publications sent through the mail.

### The Utica Tuberculosis Pavilion

THE Physicians and the Utica branch of the State Charities Aid Association held a joint meeting recently, to appoint a committee to aid in the selection of a suitable site, and to act in co-operation with the aldermen. Messrs. J. Depuyster Lynch and E. D. Ibbottson, were appointed to represent the Association, Drs. F. D. Crim andC. Gray Capron, to represent the medical profession.

Considerable difference of opinion existed as to the proper location of the hospital, our Associate Editor, Dr. Fayette H. Peck, speaking for those favoring the General Hospital Grounds; Dr. D. L. McNamara for those who preferred the more distant Dwyer site. In order not to delay action, both sides agreed not to press the choice of site.

The profession of Buffalo, having lately had a very similar experience with regard to tuberculosis, and acute contagious diseases can appreciate the problem before the citizens and profession of Utica. We trust that a satisfactory solution will be reached more promptly than has been the case in Buffalo and that a site on which all can concur, will be selected.

### A Campaign Against Beats

THE National Association of Credit Men, at the meeting of its officers in Rochester, decided to raise a million dollars, at the rate of a hundred thousand a year, for the purpose of prosecuting dead beats. The expense amounts to only about $7, for each firm represented in the membership.

This is a movement with which the medical profession will sympathize. Perhaps it will even be worth while and practicable to coöperate in some way. At any rate, the whole country will gain by it. The tax which the dead beat places on the citizen is greater than that imposed by all branches of government—at least for the average professional and business man. It is greater than the loss from honest thieves, robbers and burglars who, at least, do something to earn a living and do not sneak behind the protection of the law. And, in the long run, the dead beat himself loses more than his victim. In fact, the greatest kindness to any young man who finds it easy to borrow or to obtain credit and hard to pay, is to bring him up short and teach him that it pays to be honest.

### Army Medical Corps Examinations

THE Surgeon General of the Army announces that preliminary examinations for the appointment of first lieutenants in the Army Medical Corps will be held on January 15, 1912, at points to be hereafter designated.

Full information concerning these examinations can be procured upon application to the "Surgeon General, U. S. Army, Washington, D. C." The essential requirements to securing an invitation are that the applicant shall be a citizen of the United States, shall be between 22 and 30 years of age, a graduate of a medical school legally authorized to confer the degree of doctor of medicine, shall be of good moral character and habits, and shall have had at least one year's hospital training as an interne, after graduation. The examinations will be held concurrently throughout the country at points where boards can be convened. Due consideration will be given to localities from which applications are received, in order to lessen the traveling expenses of applicants as much as possible.

The examination in subjects of general education (mathematics, geography, history, general literature, and Latin) may be omitted in the case of applicants holding diplomas from reputable literary or scientific colleges, normal schools or high schools, or graduates of medical schools which require an entrance examination satisfactory to the faculty of the Army Medical School.

In order to perfect all necessary arrangements for the examination, applications must be complete and in possession of The Adjutant General at least three weeks before the date of examination. Early attention is therefore enjoined upon all intending applicants. There are at present sixty-four vacancies in the Medical Corps of the Army.

